# Utility of the EQUAL Candida Score for Assessing Adherence to Candidemia Management Guidelines: A Single-Center Experience

**DOI:** 10.7759/cureus.99011

**Published:** 2025-12-11

**Authors:** Kai Yan Chua, Kok Tong Tan, Wye Hong Leong, Wee Fu Gan

**Affiliations:** 1 Infectious Diseases Unit, Department of Medicine, Melaka Hospital, Melaka, MYS

**Keywords:** candidemia, equal candida score, guideline adherence, mortality, sepsis

## Abstract

Background: Candidemia remains challenging, with high morbidity and mortality. The association between clinical characteristics, disease severity, infectious disease consultation, and the EQUAL *Candida* Score was investigated.

Materials and methods: We conducted a retrospective cohort study of hospitalized adults with candidemia at Melaka Hospital from January 1, 2020, to December 31, 2024. Demographics, comorbidities, clinical characteristics, microbiological descriptions, clinical outcomes, and treatment details were analyzed.

Results: A total of 115 cases of candidemia were included in this study. The median EQUAL *Candida* Score was 12 (interquartile range (IQR) 3) for patients with central venous catheters (CVC) and 11 (IQR 2) for those without CVC. Of 115 subjects, 81 (70.4%) adhered to the candidemia guidelines, with a median EQUAL *Candida* Score of 14 (range 13-15) in the CVC group and 12 (range 11-13) in the non-CVC group (p < 0.001). However, the overall 90-day mortality rate was alarmingly high at 62%, with the adherent group experiencing an even greater rate of 64.2% when compared with the non-adherent group (p = 0.587). The regression analysis identified key risk factors significantly contributing to the high mortality rate, including severe sepsis, the need for critical care admissions, mechanical ventilation use, the absence of infectious disease consultations, the acquisition of *Candida tropicalis*, and inadequate antifungal therapy.

Conclusions: Despite the high overall mortality rate in patients with candidemia, most patients in our study had a high EQUAL *Candida* Score, indicating good adherence to the management guidelines for candidemia. Our findings suggest that illness severity, adequacy of antifungal therapy, and timely involvement in infectious diseases strongly influence patient outcomes. Early recognition and prompt, appropriate management remain essential to improving survival.

## Introduction

Candidemia, defined as the isolation of *Candida* spp. from any blood culture drawn peripherally or via central lines, is a significant form of invasive candidiasis. Notably, it is associated with a high case-fatality rate, reaching up to 47% [1]. Most candidemia cases are caused by *Candida albicans*, followed by *Candida glabrata*, *Candida tropicalis*, and *Candida parapsilosis*, with the prevalence of these species varying based on patient population and geographic area. Several risk factors predict candidemia, including immunocompromising states, prolonged exposure to broad-spectrum antimicrobial therapy, the use of central venous catheters (CVCs), hemodialysis, mechanical ventilation, neutropenia, and abdominal surgery. The treatment for candidemia is guided by evidence-based guidelines, with a strong emphasis on achieving adequate source control.

The EQUAL *Candida* Score, introduced by the European Confederation of Medical Mycology in 2018, primarily incorporates recommendations from two key candidemia guidelines: the European Society for Clinical Microbiology and Infectious Diseases Guideline and the Infectious Diseases Society of America Guideline [2-7]. Additionally, it serves as a tool for measuring the quality of clinical management related to candidemia. By benchmarking appropriate management practices across healthcare facilities, the EQUAL *Candida* Score helps identify best practices for effectively tackling candidemia.

Thus far, there is no single center in Malaysia that is evaluating the use of the EQUAL *Candida* Score to assess the clinical management of candidemia in healthcare facilities. Thus, we aimed to determine the extent of adherence to these guidelines by utilizing the EQUAL *Candida* Score and to examine its association with patient outcomes.

## Materials and methods

Methodology

This single-center retrospective cohort study was conducted at Melaka Hospital, a 1100-bed tertiary care center in Melaka, Malaysia. All hospitalized adults aged ≥18 years with a first episode of blood cultures positive for *Candida* species from January 1, 2020, to December 31, 2024, were included. Exclusion criteria included individuals who died within 48 hours of the time of blood cultures first isolating *Candida* species, those with incomplete records with missing data, pregnant women, and children under 18 years of age. Additionally, individuals receiving palliative care who were untreated with antifungal therapy were also excluded. Our study was approved with a waiver of informed consent. Medical records and electronic databases were assessed to collect demographic information, medical history (including comorbidities), microbiological characteristics, and options of antifungal therapy. Additional data were obtained from the National Registration Department to verify patient survival status in this study accurately.

The EQUAL *Candida* Score is designed to assess adherence to recommended diagnostic, therapeutic, and follow-up steps in the management of candidemia (Table 1). In our study, it was utilized to evaluate the alignment between clinical practices and guidelines. Patients without a CVC can reach a maximum score of 19 points, whereas patients with a CVC can reach a maximum score of 22 points. The EQUAL *Candida* Score cut-off values were estimated based on the median score observed in each subgroup. The medians of the EQUAL *Candida* Score were 13 and 11 in patients with and without a CVC, respectively. These cut-offs were chosen to divide patients into adherence (greater than or equal to the median) and non-adherence (less than the median) groups, to ensure equal distribution and to make comparisons of outcomes relevant to real-life adherence tendencies, which were observed in our cohort.

**Table 1:** EQUAL Candida Score: components and corresponding point allocation for candidemia management Adapted from [2]. Permission obtained from the copyright owner. CVC: central venous catheter

Category	Action/test	Points
Diagnosis	Initial blood culture (40 mL)	3
Diagnosis	Species identification	3
Diagnosis	Susceptibility testing	2
Diagnosis	Echocardiography	1
Diagnosis	Ophthalmoscopy	1
Treatment	Echinocandin treatment	3
Treatment	Step down to fluconazole depending on the susceptibility result	2
Treatment	Treatment for 14 days after the first negative follow-up culture	2
Treatment	CVC removal ≤24 hours from diagnosis (CVC carriers only)	3
Treatment	CVC removal >24 <72 hours from diagnosis (CVC carriers only)	2
Follow-up	Follow-up blood culture (at least one per day until negative)	2

Statistical analysis

Continuous variables were described as medians and interquartile range (IQR) and compared using either Student’s t-test or the Mann-Whitney U test. Categorical variables were presented as absolute frequencies and compared using the chi-square test or Fisher’s exact test. Odd ratios were calculated using binary logistic regression to quantify the relationship between independent variables and 90-day mortality. All statistical analyses were performed using SPSS Statistics version 27.0 (IBM Corp. Released 2020. IBM SPSS Statistics for Windows, Version 27.0. Armonk, NY: IBM Corp.).

## Results

**Figure 1:**
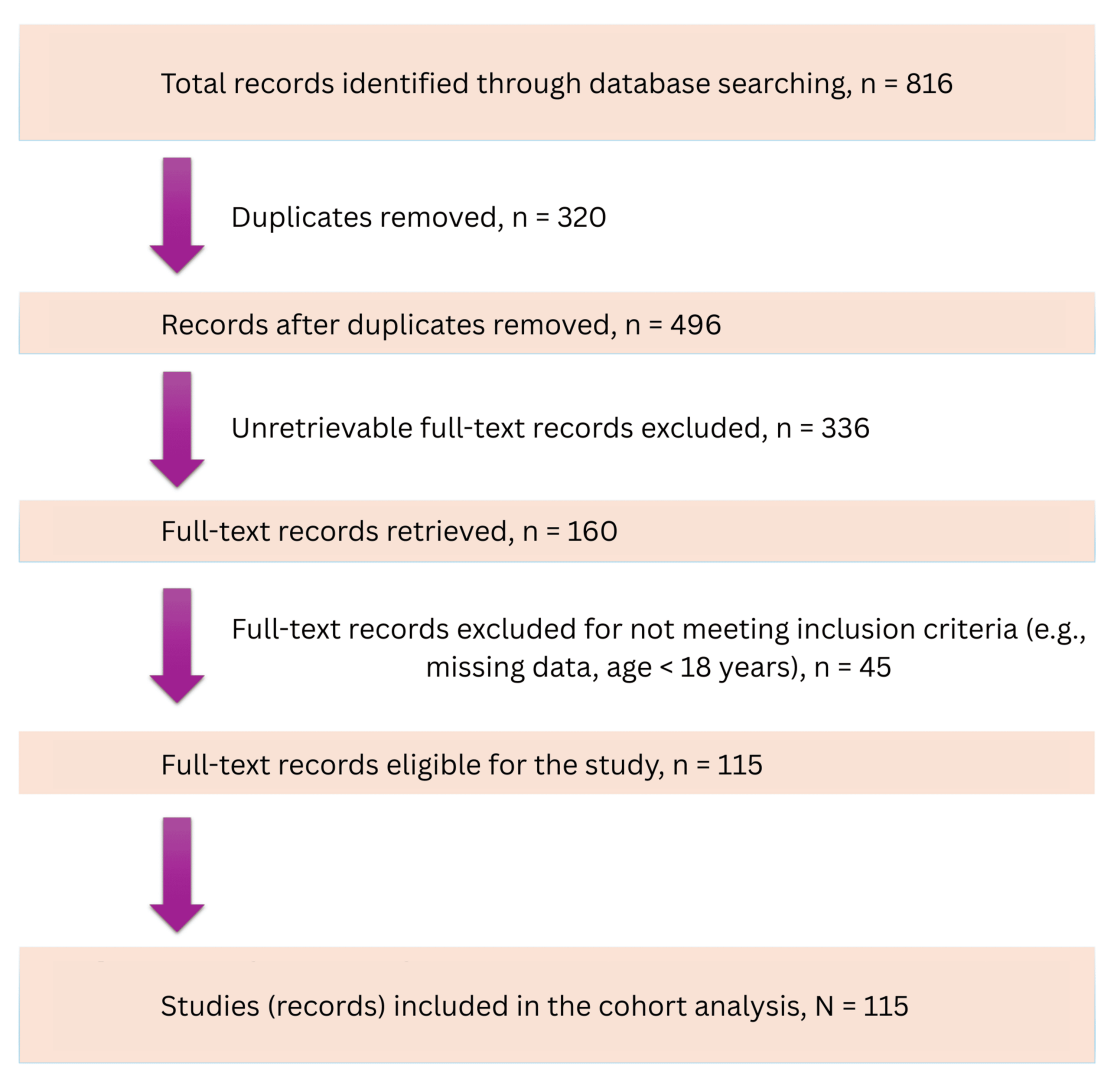
Study selection flowchart for candidemia cohort analysis Flowchart depicting the selection process for candidemia cohort analysis. From 816 records initially identified, 320 duplicates were removed. Of the 496 remaining, 336 could not be retrieved in full text. The remaining 160 were assessed in detail, with 45 excluded due to reasons such as missing data or patient age under 18 years. A total of 115 records were ultimately included in the cohort analysis.

We identified 496 patients who had candidemia at Melaka Hospital during the study period. Nevertheless, 381 patients were excluded from the study (Figure 1). Ultimately, 115 patients met the study inclusion criteria. Table 2 demonstrates the comparative characteristics of these 115 enrolled patients. The baseline characteristics, including patient demographics and comorbidities, were similar across both groups. Among 115 patients, 81 (70.4%) adhered to the guideline management for candidemia, achieving an EQUAL *Candida* Score ≥13 for those with CVC and ≥11 for those without CVC.

**Table 2:** Comparison of clinical characteristics, risk factors, guideline adherence, and mortality outcomes between patients with and without CVC by EQUAL Candida Score adherence at a Malaysian tertiary hospital (n = 115) EQUAL *Candida* Score adherence thresholds were defined as ≥13 for patients with CVC and ≥11 for those without CVC. Comparisons were made between adherent and non-adherent groups within each subgroup. Variables compared include demographic characteristics, comorbidities, clinical severity, *Candida* species distribution, antifungal treatment patterns, and adherence to key guideline components. Statistical significance was determined using χ² or Fisher’s exact test for categorical variables and independent-samples t-test or Mann-Whitney *U* test for continuous variables. CVC: central venous catheter, χ²: chi-square test, t: independent-samples t-test, U: Mann-Whitney U test, df: degrees of freedom, SD: standard deviation, IQR: interquartile range, TPN: total parenteral nutrition, TTE: transthoracic echocardiogram, TOE: transoesophageal echocardiogram

Variable	With CVC (n = 63)				Without CVC (n = 52)			
									Adhere (≥13) (n = 39)	Non-adhere (≤12) (n = 24)	p-value	Test statistic (df)	Adhere (≥11) (n = 42)	Non-adhere (≤10) (n = 10)	p-value	Test statistic (df)
	Age, median (SD)	56.2 (14.6)	58.9 (13.7)	0.473	t(61) = 0.72	61.7 (14.7)	53.5 (15.6)	0.124	t(50) = -1.57
Male	28 (66.7%)	14 (33.3%)	0.271	χ²(1) = 1.212	24 (57.1%)	4 (40%)	0.328	χ²(1) = 0.955
Female	11 (52.4%)	10 (47.6%)	0.271	χ²(1) = 1.212	18 (42.9%)	6 (60%)	0.328	χ²(1) = 0.955
Main comorbidities								
Diabetes mellitus	21 (53.8%)	15 (62.5%)	0.5	χ²(1) = 0.454	26 (61.9%)	7 (70%)	0.633	χ²(1) = 0.228
Immunocompromised state	9 (23.1%)	1 (4.2%)	0.046	χ²(1) = 3.979	6 (14.3%)	1 (10%)	0.721	χ²(1) = 0.127
Chronic kidney disease	11 (28.2%)	13 (54.2%)	0.039	χ²(1) = 4.246	8 (19%)	1 (10%)	0.497	χ²(1) = 0.462
Chronic liver disease	1(2.6%)	0 (0%)	1	χ²(1) = 0.625	3 (7.1%)	2 (20%)	0.242	χ²(1) = 1.536
Cardiovascular disease	10 (25.6%)	6 (25%)	0.955	χ²(1) = 0.003	5 (11.9%)	0 (0%)	0.251	χ²(1) = 1.317
Clinical condition								
Mechanical ventilation	20 (51.3%)	9 (37.5%)	0.287	χ²(1) = 1.136	20 (47.6%)	5 (50%)	0.892	χ²(1) = 0.018
Critical care admission	22 (56.4%)	11 (45.8%)	0.414	χ²(1) = 0.666	25 (59.5%)	6 (60%)	0.978	χ²(1) = 0.001
Requirement of TPN	2 (5.1%)	0 (0%)	0.521	χ²(1) = 1.271	0 (0%)	0 (0%)	-	-
Septic shock/severe sepsis	36 (92.3%)	19 (79.2%)	0.128	χ²(1) = 2.314	36 (85.7%)	8 (80%)	0.653	χ²(1) = 0.203
Neutropenia	2 (5.1%)	2 (8.3%)	0.632	χ²(1) =0.257	3 (7.1%)	1 (10%)	0.761	χ²(1) = 0.093
*Candida* species								
Candida albicans	9 (23.1%)	4 (16.7%)	0.541	χ²(1) = 0.373	10 (23.8%)	5 (50%)	0.1	χ²(1) = 2.699
Candida tropicalis	9 (23.1%)	3 (12.5%)	0.299	χ²(1) = 1.078	16 (38.1%)	2 (20%)	0.28	χ²(1) = 1.169
Candida glabrata	8 (20.5%)	5 (20.8%)	0.976	χ²(1) = 0.001	9 (21.4%)	1 (10%)	0.41	χ²(1) = 0.679
*Candida parapsilosis* complex	10 (25.6%)	8 (33.3%)	0.512	χ²(1) = 0.431	3 (7.1%)	0 (0%)	1	χ²(1) = 0.758
Other *Candida* species	2 (5.1%)	4 (16.7%)	0.19	χ²(1) = 2.296	1 (2.4%)	2 (20%)	0.091	χ²(1) = 4.612
Antifungal treatment								
No antifungal treatment	0 (0%)	4 (16.7%)	0.018	χ²(1) = 6.941	0 (0%)	6 (60%)	<0.001	χ²(1) = 28.487
Fluconazole	29 (74.4%)	17 (70.8%)	0.759	χ²(1) = 0.094	29 (69%)	4 (40%)	0.086	χ²(1) = 2.939
Polyene	2 (5.1%)	0 (0%)	0.521	χ²(1) =1.271	2 (100%)	0 (0%)	1	χ²(1) = 0.495
Echinocandins	8 (20.5%)	3 (12.5%)	0.416	χ²(1) = 0.662	12 (28.6%)	0 (0%)	0.054	χ²(1) = 3.714
Guideline adherence								
Susceptibility testing done	38 (97.4%)	13 (54.2%)	<0.001	χ²(1) = 18.039	42 (100%)	8 (80%)	0.003	χ²(1) = 8.736
Adequate antifungal treatment (≥14 days)	31 (79.5%)	8 (33.3%)	<0.001	χ²(1) = 13.42	20 (47.6%)	0 (0%)	0.005	χ²(1) = 7.738
blood culture surveillance	39 (100%)	19 (79.2%)	0.003	χ²(1) = 8.825	37 (88.1%)	5 (50%)	0.006	χ²(1) = 7.546
CVC removal (if applicable)	38 (97.4%)	19 (79.2%)	0.016	χ²(1) = 5.755	N/A	N/A	N/A	N/A
Referral to ophthalmology	24 (61.5%)	4 (16.7%)	<0.001	χ²(1) = 12.115	24 (57.1%)	1 (10%)	0.007	χ²(1) = 7.191
TTE/TOE done	39 (100%)	19 (79.2%)	0.003	χ²(1) = 8.825	39 (92.9%)	5 (50%)	0.004	χ²(1) = 11.396
EQUAL *Candida* Score								
Median (IQR)	14 (13–15)	11 (10-12)	<0.001	U = 0.000	12 (11-13)	8 (8-10)	<0.001	U = 0.000
90-day mortality outcome								
Deceased	22 (56.4%)	14 (58.3%)	0.881	χ²(1) = 0.022	30 (71.4%)	6 (60%)	0.482	χ²(1) = 0.495

Of 115 patients, antifungal susceptibility testing was performed on 101 individuals (88%) with a strong adherence rate of 98.8% (p < 0.001), follow-up blood cultures in 100 patients (87%) with a 93.8% adherence rate (p = 0.001), ophthalmology referral in 53 patients (46.1%) with a 59.3% adherence rate (p < 0.001), and echocardiography in 102 patients (88.7%) with a 96.3% adherence rate (p < 0.001). Of 63 patients with CVCs, 57 (90.5%) had their CVCs removed, yielding an adherence rate of 97.4% (p = 0.03). Among the 34 patients classified as non-adherent, 10 (29.4%) received no antifungal treatment in the CVC subgroup, and six in the non-CVC subgroup. Of 115 patients, 51 patients (44.3%) received adequate antifungal treatment for ≥14 days, corresponding to a 63% adherence rate (p < 0.001).

The overall 90-day mortality rate was as high as 63%. Among 81 patients who adhered to the guidelines, 52 (64.2%) died (p = 0.587). Of 34 patients with low EQUAL *Candida* Score, 20 (58.8%) died. As shown in Table 3, several key factors predicting 90-day mortality included mechanical ventilation use (OR 0.386, p = 0.018, CI 95% 0.175-0.85), critical care admission (OR 0.296, p = 0.296, CI 95% 0.135-0.652), severe sepsis (OR 0.1, p < 0.001, CI 95% 0.027-0.378), *Candida tropicalis* acquisition (OR 0.247, p = 0.009, CI 95% 0.086-0.708), inadequate antifungal treatment less than 14 days (OR 0.171, p = 0.001, CI 95% 0.073-0.403), and absence of infectious disease consultations (OR 0.187, p < 0.001, CI 95% 0.082-0.43). In comparison to the non-adherent group, the adherent group had mechanical ventilation (74.1% vs. 25.9%, p = 0.421), critical care admissions (73.4% vs. 26.6%, p = 0.429), severe sepsis (72.7% vs. 27.3%, p = 0.18), and *Candida tropicalis* acquisition (83.3% vs. 16.7%, p = 0.072).

**Table 3:** Comparison of clinical characteristics, risk factors, and guideline adherence between survivors and non-survivors of candidemia at a Malaysian tertiary hospital (n = 115) This table compares clinical characteristics, risk factors, *Candida* species, antifungal therapy, and guideline adherence measures between survivors and non-survivors of candidemia at 90 days. Survival status was determined at 90 days post-index candidemia episode. EQUAL *Candida* Score thresholds were evaluated separately for patients with and without CVC. Statistical tests included χ² or Fisher’s exact test and Mann-Whitney *U* test as appropriate. χ²: chi-square test, df: degrees of freedom, IQR: interquartile range, CVC: central venous catheter, TPN: total parenteral nutrition, TTE: transthoracic echocardiogram, TOE: transoesophageal echocardiogram

Variable	Total (N = 115, %)	Survivors (N = 43, %)	Non-survivors (N = 72, %)	p-value	Test statistic (*df*)
Mechanical ventilation	54 (47)	14 (25.9)	40 (74.1%)	0.017	χ²(1) = 5.717
Critical care admission	64 (55.7)	16 (25)	48 (75%)	0.002	χ²(1) = 9.465
Septic shock/severe sepsis	99 (86.1)	30 (30.3)	69 (69.7%)	<0.001	χ²(1) = 15.272
Candida tropicalis	30 (26.1)	5 (16.7)	25 (83.3%)	0.006	χ²(1) = 7.447
Susceptibility testing done	101 (87.8)	35 (34.7)	66 (65.3%)	0.103	χ²(1) = 2.656
Adequate antifungal treatment (≥14 days)	59 (51.3)	33 (55.9)	26 (44.1%)	<0.001	χ²(1) = 17.792
Follow-up blood culture taken	100 (87)	38 (38)	62 (62%)	0.728	χ²(1) = 0.121
CVC removal (if applicable)	57 (49.6% of 63)	25 (43.9% of 27)	32 (56.1% of 36)	0.361	χ²(2) = 2.035
Referral to ophthalmology	53 (46.1)	24 (45.3)	29 (54.7)	0.106	χ²(1) = 2.615
TTE/TOE performed	102 (88.7)	36 (35.3)	66 (64.7)	0.193	χ²(1) = 1.695
IDC	42 (36.5)	26 (61.9)	16 (38.1)	<0.001	χ²(1) = 16.984
Non-IDC	73 (63.5)	17 (23.3)	56 (76.7)	<0.001	χ²(1) = 16.984

Compared with the non-IDC arm, the IDC arm exhibited a higher usage of echinocandins (69.6% vs. 30.4%, p < 0.001) and polyene (100% vs. 0%, p = 0.007). The IDC group also had a greater portion of patients receiving adequate antifungal treatment for ≥14 days (49.2% vs. 50.8%, p = 0.004). Additionally, the IDC arm had more follow-up blood cultures performed (95.2% vs. 82.2%, p = 0.045) and a higher rate of regular ophthalmology referrals (58.5% vs. 41.5%, p < 0.001). In the IDC group, the median EQUAL *Candida* Score was higher (13 vs. 12, p < 0.001), and 90-day mortality was lower (22.2% vs. 77.8%, p < 0.001). In other words, a higher survival rate (60.5% vs. 39.5%, p < 0.001) was observed in the IDC arm.

## Discussion

To our knowledge, this is the first paper in Malaysia to examine adherence to candidemia guidelines using the EQUAL *Candida* Score. We found that up to 71% of patients had a high EQUAL *Candida* Score, especially amongst patients receiving IDC, indicating better adherence to guidelines. However, we observed a high 90-day mortality in the adherent arm, with most of these patients being critically ill, experiencing severe sepsis, and requiring critical care admissions on mechanical ventilation. Additionally, the predictors of mortality were the acquisition of *Candida tropicalis* and the absence of IDC.

The EQUAL *Candida* Score, developed in 2018, summarizes the recommendations for managing patients with candidemia. Components incorporated include diagnostic methods (ophthalmoscopy, echocardiography, species identification), follow-up procedures (blood culture re-testing), and treatment strategies (echinocandin, optimization of antifungal therapy, CVC removal) [2]. It serves as a tool for monitoring adherence to guidelines, facilitating decision-making, and improving the quality of care. Previous studies have shown that higher EQUAL *Candida* Scores are significantly correlated with better clinical outcomes. For every point decline in the EQUAL *Candida* Score, the risk of mortality increases, indicating poor adherence to guideline recommendations. Interestingly, Hoenigl et al. reported a 9% increase in mortality risk among patients with a CVC and an 8% increase among patients without a CVC as the EQUAL *Candida* Score progressively decreased [8].

Nevertheless, in our study, we observed a strikingly high mortality rate among patients in the adherent group with an elevated EQUAL *Candida* Score. This alarming trend highlights that the majority of these patients were severely ill and required mechanical ventilation in intensive care units. Of note, the 90-day mortality of 63% observed in our study indicates that candidemia remains a significant threat to patients. Previous studies have reported mortality rates associated with candidemia ranging from 26% to 71%, which can be reduced by up to 50% with the prompt initiation of appropriate therapy [1,7-9]. Consistent with Hoenigl et al.’s study, we identified several independent predictors of mortality, including severe sepsis requiring critical care, mechanical ventilation, acquisition of *Candida tropicalis*, inadequate antifungal therapy, and absence of IDC [8]. Notably, the presence of septic shock and the need for mechanical ventilation highlight the severity of infection and severe physiological changes involved, resulting in a high case-fatality rate.

Our study revealed that IDC was associated with a higher EQUAL *Candida* Score and lower mortality in patients with candidemia. Rieg and Küpper discovered favorable effects of IDC in critically ill patients with complex infections, including endocarditis, with higher rates of appropriate therapy and improved clinical outcomes [10]. Similarly, Mejia-Chew et al. reported that prompt IDC improved survival rates [1]. Additionally, the ID team often facilitates the timely identification and control of infectious sources. Of note, we reported that the IDC group frequently received ophthalmological assessments to evaluate endophthalmitis. This practice aligns with IDSA guidelines, which recommend that all non-neutropenic adults with candidemia should receive ophthalmological evaluation [7]. Conversely, Ryder et al. reported that IDC was not associated with better clinical outcomes in candidemia [11].

The emergence of non-albicans *Candida*, mainly *Candida tropicalis*, significantly affects clinical outcomes and increases hospital expenditure. Interestingly, Tan et al. reported a shift in the causes of candidemia toward non-albicans *Candida* spp., with a more resistant pattern [12]. Additionally, there was a 6.1% increase in the isolation of *Candida tropicalis* from blood cultures, rising from 14.5% in 2020 to 20.6% in 2021. Alarmingly, *Candida tropicalis* exhibits a high species-dependent mortality ranging from 55% to 64% [13,14]. Hoenigl et al. identified that *Candida tropicalis* was a significant predictor of mortality in the setting of candidemia [8]. Keighley et al. reported high resistance rates to triazoles (including fluconazole, itraconazole, and voriconazole) up to 40-80% [14]. Conversely, *Candida tropicalis* isolates exhibited high susceptibility rates to the echinocandins (less than 1%), polyenes (0%), and flucytosine (less than 5%) [14].

In our study, all *Candida* spp. were susceptible to polyenes and echinocandins (with limited activity against micafungin and anidulafungin). Guidelines recommend echinocandin as the preferred first-line agent for critically ill patients with candidemia [2,7]. Huang et al. described that up to 50.7% of critically ill patients with candidemia were treated with echinocandins [15]. However, echinocandin use in our study was significantly lower in the non-IDC group. Additionally, fluconazole was often chosen as the first-line treatment in the non-IDC cohort, given that *Candida​​​​​​​ albicans* was the most prevalent *Candida* spp. in our cohort. Interestingly, a proportion of patients (10%) in the non-IDC group with candidemia did not receive any antifungal treatment. One common explanation for withholding antifungal therapy could be a scarcity of familiarity with managing candidemia. Additionally, positive blood cultures might sometimes be regarded as contaminants, leading to false-positive results.

On the other hand, patients with candidemia who had IDC received better initial antifungal therapy selection and longer duration of therapy. Interestingly, Takakura et al. reported that the appropriateness of antifungal treatment was higher among patients with IDC than among those without it [16]. Although the antifungal options were similar and available to both groups in our study, the IDC group received a significantly longer course, more than 14 days. The extended duration of antifungal therapy may be due to the complexity of cases involving IDC, which are often characterized by the dissemination of infective foci, documented clearance on blood culture, or appropriate source control of infections established on the first day of antifungal therapy.

This study has several limitations. First, due to its retrospective design, selection and information bias may have been introduced, as the findings relied on the completeness and accuracy of the available medical records. Additionally, the results of our study may not be generalizable to other medical facilities, which may have different resource availability, clinical approaches, or patient demographics. Furthermore, while the EQUAL *Candida* Score is applicable for demonstrating adherence to guidelines, it does not fully capture the complexities of clinical decision-making, e.g., the need for a personalized approach in managing complex patients or the exclusion of specific interventions. Moreover, the small sample size limited the statistical power of the subgroup analysis. Finally, there is a risk of selection bias, as some cases were excluded due to missing data or irretrievable records, potentially underestimating the true burden of non-adherence or mortality.

## Conclusions

We suggest that the EQUAL *Candida* Score be considered a valuable tool for monitoring the quality of care and guiding future improvements in candidemia management. Despite good guideline adherence in our setting, the overall 90-day mortality rate remains high. The independent predictive factors with increased 90-day case fatality include severe sepsis, use of mechanical ventilation, critical care admissions, acquisition of *Candida tropicalis*, and the lack of IDC. We believe that early recognition of patients requiring antifungal therapy and prompt treatment of sanctuary-site infections helps reduce the case-fatality rate. Further studies are anticipated to explore the challenges that hinder the clinical management of patients with candidemia and to identify solutions.
